# Exploring the factors associated with professional and non-professional dancer well-being: a comprehensive systematic review

**DOI:** 10.3389/fpsyg.2025.1644253

**Published:** 2025-08-26

**Authors:** Haiyan Yu, Eng Wah Teo, Chai Chen Tan, Jindong Chang, Shenghui Liu

**Affiliations:** ^1^Faculty of Sports and Exercise Science, Universiti Malaya, Kuala Lumpur, Malaysia; ^2^School of Physical Education, Fuyang Normal University, Fuyang, China; ^3^Faculty of Creative Arts, Universiti Malaya, Kuala Lumpur, Malaysia; ^4^School of Physical Education, Southwest University, Chongqing, China

**Keywords:** well-being, dancers, eudaimonic well-being, hedonic well-being, mental health, systematic review

## Abstract

**Background:**

Dance is a physically and psychologically demanding activity that can significantly affect dancers’ overall well-being. While interest in this area has increased, no comprehensive systematic review has synthesized existing findings across diverse populations and contexts. This study addresses this gap by reviewing how dancers’ well-being has been conceptualized and measured, identifying key associated factors, and evaluating available interventions.

**Methods:**

Following PRISMA guidelines, 18 peer-reviewed studies published from 1984 to November 2024 were included, sourced from Web of Science, Scopus, PubMed, SPORTDiscus, and manual searches. The Mixed Methods Appraisal Tool (MMAT) was used to assess the risk of bias.

**Results:**

The synthesized evidence reveals a growing trend toward adopting eudaimonic and multidimensional frameworks in the conceptualization of dancer well-being, with the Positive and Negative Affect Schedule being the most frequently employed instrument to measure dancers’ well-being. Dancers’ well-being is significantly associated with multiple factors, including demographic factors, motivational climate, psychological factors, as well as organizational stressors and resources. Notably, only one study employed a randomized controlled trial design.

**Conclusion:**

This review also identifies several important areas for future research, including the adoption of longitudinal and mixed-methods designs, the development of validated and dancer-specific measures of well-being, and the design of interventions applicable to dancers across a broader range of dance genres, professional levels, and underrepresented groups, such as male dancers and individuals from diverse cultural contexts. These findings provide an integrated understanding of the key psychological factors, theoretical models, and methodological approaches shaping dancers’ well-being, offering a foundation for future targeted interventions and research.

**Systematic review registration:**

CRD420251003173, https://www.crd.york.ac.uk/PROSPERO/view/CRD420251003173.

## Introduction

Well-being is a universally recognized priority (Heimburg et al., 2022). As a form of artistic expression, dance is widely acknowledged for its positive impact on human well-being (Chappell et al., 2021; Stutesman et al., 2025). However, recent research highlights a concerning prevalence of mental health issues among professional and collegiate dancers (Michaels et al., 2023). Growing attention has been given to the challenges of sustaining dancers’ well-being, underscoring the need for a deeper understanding of its conceptualization and associated factors (Collard-stokes and Irons, 2022).

Historically, wellbeing has been conceptualized through two distinct perspectives: the hedonic and the eudaimonic approaches (Ryan and Deci, 2001; Delle fave et al., 2011; Ryff, 2013). Hedonic well-being emphasizes subjective experiences of happiness and life satisfaction (Diener, 2000). This perspective underlies the concept of subjective well-being (SWB), which comprises life satisfaction, positive affect, and negative affect (Diener and Sim, 2024). In contrast, eudaimonic well-being refers to optimal psychological functioning and the realization of personal potential (Ryff and Singer, 2008). Ryff’s psychological well-being model conceptualizes this through six dimensions: autonomy, positive relationships, environmental mastery, self-acceptance, purpose in life, and personal growth (Ryff, 2013). Complementing this, self-determination theory (SDT) focuses on the satisfaction of three basic psychological needs: autonomy, competence, and relatedness, which are essential for sustained well-being (Ryan and Deci, 2000). Despite the significance of well-being, no universally accepted definition exists within the social sciences (Huppert and So, 2013), and researchers continue to debate its conceptualization and measurement.

Recent literature suggests that well-being should be considered both as a global construct, which encompasses hedonic and eudaimonic perspective (Henderson and Knight, 2012; VanderWeele and Johnson, 2025), and in context-specific terms, where domain-related experiences and goals shape an individual’s well-being (Lundqvist, 2011). In this light, wellbeing can be understood as a multifaceted satisfaction with life arising from integrating psychological, social, and physical elements.

While well-being is important for everyone, it is especially critical for dancers. Dancing merges athletic performance with artistic expression within the performing arts, placing unique demands on the body and mind (McEwen and Young, 2011; Uršej and Zaletel, 2020). Physically, dancers face rigorous training schedules, repetitive movements, and high expectations for technical precision, often leading to overuse injuries, fatigue, and chronic pain (Michaels et al., 2023; Nicholas and Grafenauer, 2023). Psychologically, dancers must navigate the constant scrutiny of their appearance and performance by teachers, choreographers, peers, and audiences (Smith et al., 2024), which can contribute to performance anxiety, body image dissatisfaction, and heightened stress (Junge and Hauschild, 2023).

A systematic review of performing artists’ occupational demands and well-being highlights that organizational and occupational stress can negatively impact well-being. In contrast, performance activities and social support may be protective or enhancing influences (Willis et al., 2019). However, their review encompassed a broad range of disciplines, including musicians,

actors, and dancers, and only two of the included studies specifically focused on dancers. This limited representation constrains the applicability of their conclusions to the dance context, which involves unique physical, psychological, and cultural demands (Bond, 2024). For example, body maturation, authoritarian teaching styles, and conformity pressures inherent in dance culture, which distinguish dancer well-being from that of other performance disciplines (Mitchell et al., 2020; Rowe et al., 2020).

In recent years, dancer-specific research has grown substantially, a new systematic review is therefore timely and necessary to synthesize this growing body of literature, clarify how dancer well-being is conceptualized and measured, as well as identify key associated factors and effective interventions would contribute to the research and practice in dancers’ mental health.

Two key gaps exist in the literature: First, in the context of dance, there is no PRISMA systematic review that comprehensively summarizes well-being research. Although prior review have explored the relationship between occupational demands and artists’ well-being, without fully examining the overall trends in dancers’ well-being (Willis et al., 2019). Second, the conceptualization and measurement of well-being in dance research remain underdeveloped (Fauntroy et al., 2020). In this sense, Paschali and Araújo (2023) highlight the need for context-specific, system-level approaches to dancer well-being, yet current evidence remains fragmented. Thus, there is a pressing need for a more integrated and systematic synthesis to inform future research and practice.

This study aims to conduct a systematic review of well-being among dancers, specifically (1) to describe the methodologies, instruments, and samples used in the studies; (2) to summarize the conceptualizations of well-being among dancers; (3) to determine factors associated with the well-being of dancers.

## Methods

### Research strategy

This review adhered to the Preferred Reporting Items for Systematic Reviews and Meta-Analyses (PRISMA) guidelines (Moher et al., 2009). We used a mixed strategy combining electronic database searches with hand searches to expand the retrieval scope. The hand search involved reviewing the reference lists of key articles and relevant reviews to identify additional studies that may not have been captured through database searching. The electronic literature search was conducted using the following databases: Web of Science, SPORTDiscus, PubMed, Scopus, and Google Scholar. Our search criteria encompassed titles, abstracts, and keywords. The following keywords were included in the search strategy: (dance* OR “dance student” OR ballet OR “jazz dance” OR “modern dance” OR “hip hop dance” OR “contemporary dance” OR “traditional dance”) AND (“well-being” OR “wellbeing”).

### Inclusion and exclusion criteria

The inclusion criteria for this systematic review were as follows: (1) Peer-reviewed journal articles published in English, a restriction commonly used to ensure consistency and minimize bias in systematic reviews (Morrison et al., 2012), additionally, a preliminary search of Chinese databases identified no eligible studies; (2) Articles from November 1984 up to November 2024; (3) Studies investigate well-being; (4) Research focusing on professional dancers (e.g., elite and vocational dancers) and non-professional dancers (e.g., dance students, university dancers, or collegiate dance students). The exclusion criteria were as follows: (1) Articles with English titles and abstracts but with non-English main text; (2) Review studies; (3) Non-journal articles, such as book chapters, conference papers, theses, or organizational reports;(4) Studies with non-dancer samples or participants only involved in short-term dance interventions, such as (Goulimaris et al., 2014);(5) Focused on the pandemic (COVID-19) on dancer’s wellbeing, such as (Kolitsida et al., 2023), which was excluded due to its context-specific nature that may limit generalizability across time and settings; (6) Quantitative studies that did not test the relationships between well-being and other variables, such as (Aujla and Needham-Beck, 2020).

### Screening process

**Figure 1:**
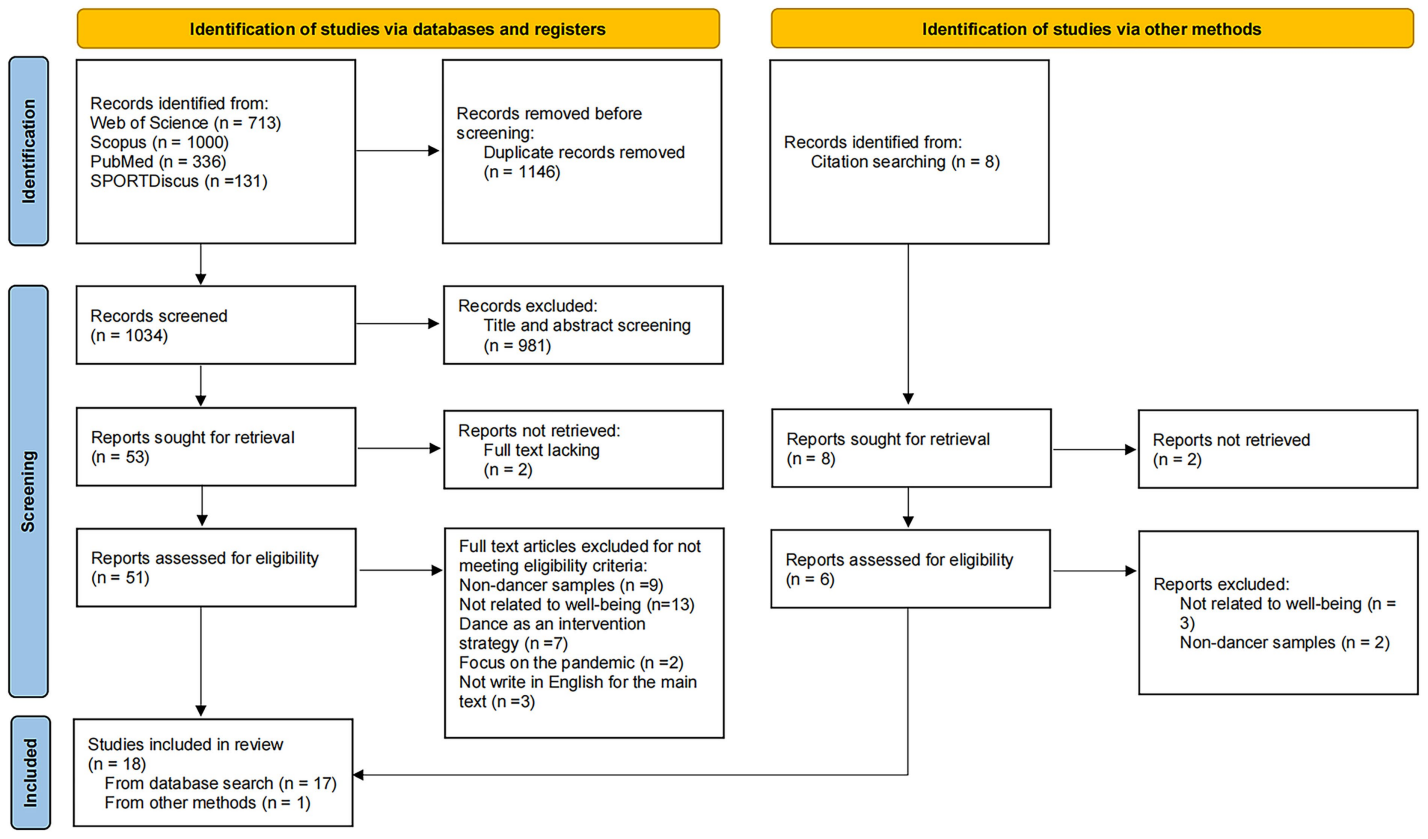
Flow diagram illustrating the screening process.

The results of the screening process are summarized in Figure 1. After completing the mixed search and identifying all eligible articles, the records were imported into Endnote. The first and second authors independently screened titles and abstracts to exclude irrelevant studies. To ensure rigor, the third and fourth authors reviewed a subset of excluded articles during the abstract and full-text screening phases. The first and second authors then independently completed the final screening and reviewed the selected studies. Any discrepancies encountered during the screening process were resolved through discussion among all authors. Interrater reliability was assessed using Cohen’s Kappa, which yielded a value of 0.91. Key information was then extracted from each study, including authors, publication year, sample characteristics, country of the study, well-being measures, study design, and main findings.

### Quality appraisal

The critical appraisal of study quality was conducted using the Mixed Methods Appraisal Tool (MMAT) (Hong et al., 2018), which has been widely applied in systematic reviews across various fields, including dance arts and sports disciplines. The MMAT assesses five study designs: qualitative research, randomized controlled trials, non-randomized studies, quantitative descriptive studies, and mixed methods. Each design is evaluated using five core criteria rated “yes,” “no,” or “cannot tell,” following two initial screening questions: “Are there clear research questions?” and “Do the collected data allow to address the research questions?” Studies that fail either screening question are considered ineligible. Each of the included articles was independently reviewed and rated by three investigators (HY, EWT, and SL) and disagreements were resolved by discussion. In this review, the MMAT was applied to qualitative, quantitative descriptive, and mixed methods studies to ensure methodological rigor and the trustworthiness of findings.

## Results

### Characteristics of included studies

Initially, an electronic search yielded 2,188 articles. After applying the exclusion criteria, 18 empirical studies remained in the final scientific studies (see Table 1). Research on well-being in the context of dance is still at a relatively early stage, with core concepts and theoretical frameworks only beginning to emerge in the past decade. The first empirical studies examining dancer well-being appeared around 2009. Since then, the number of publications has gradually grown, particularly after 2013, with a peak in output observed around 2020.

**Table 1:** Study characteristics and main findings.

ID	Author (Year)	Sample N (F/M/unspecified) Age range Mage ± SD (range)	Country	Study design	Measurements of well-being/themes	Main findings (factors associated with well-being of dancers)
1	Quested and Duda (2009)	Hip hop dancers 59 (38/21/0) - 20.29 ± 2.99	UK	Quantitative: cross-sectional survey	20-items PANAS	Satisfaction of the need for competence was positively associated with positive affect, and negatively linked to negative affect. Competence need satisfaction significantly mediated the relationship between a perceived task-involving climate and positive and negative affective states.
2	Quested and Duda (2010)	Vocational dancers 392 (293/96/3) - 18.67 ± 2.26	UK	Quantitative: cross-sectional survey	20-items PANAS	Task involvement, autonomy support, and basic need satisfaction positively predicted positive affect, while competence and relatedness negatively predicted negative affect. Ego involvement positively predicted negative affect.
3	Cahalan and O'Sullivan (2013)	Current and retired professional Irish dancers 165 (103/62/0) 18–40+ -	Irish	Qualitative: Open-ended questions; focus group interviews	Themes: positive aspects negative aspects	Ninety-four percent of surveyed PIDs and 100% of focus group participants stated that they would recommend a career in professional Irish dance. Positive aspects of dance as a job related to global travel, hobby as a career, financial benefits, friendships, physical fitness. Negative aspects related to job insecurity, injury, psychological consequences.
4	Padham and Aujla (2014)	Professional dancers 92 (69/23/0) 19–35 27.03 ± 3.84	USA, UK, Canada, Japan, Belgium, Switzerland, France, Australia, and Bulgaria.	Quantitative: cross-sectional survey	EAT-26; RSES; PI	Harmonious passion positively predicted self-esteem, while obsessive passion predicted self-evaluative perfectionism, conscientious perfectionism, and disordered eating attitudes. Additionally, self-evaluative perfectionism mediated the relationship between obsessive passion and disordered eating attitudes.
5	Stark and Newton (2014)	Adolescent dancers 83 (83/0/0) 15–18 16.28 ± 0.93	USA	Quantitative: cross-sectional survey	20-items PANAS; BESAA; IPPA	A positive climate (low ego-involvement, high task-involvement and caring) leads to positive affect, body esteem, and more friends than a mixed climate (high ego-involvement, low task-involvement and caring).
6	Draugelis et al. (2014)	University dance students 182 (157/25/0) 18–43 20.4 ± 3.2	USA	Quantitative: cross-sectional survey	AWQ	Dance self-concept and task motivation climate positively predicted well-being elements: vigor, enthusiasm, confidence, and dedication. Ego climate showed no relation to well-being.
7	Hancox et al. (2017)	Vocational dancers 135 (110/21/4) - 15.57 ± 2.48	UK	Quantitative: diary methodology	PANAS	Basic need satisfaction mediated the relationship between empowering environments and positive affect, while basic need thwarting mediated the relationship between disempowering environments and negative affect.
8	Behera and Rangaiah (2017)	Dancers 292 (127/165/0) - 32.43 ± 5.22	Indian	Quantitative: cross-sectional survey	LSS	Emotional maturity has a significant positive impact on life satisfaction. Self-esteem moderates the relationship between emotional maturity and life satisfaction.
9	Balk et al. (2018)	Dance students 80 (55/25/0) - 19.3 ± 1.3	Netherlands	Quantitative: Cross-sectional survey	I-PANAS-SF	Emotional demands were negatively related to positive affect. Emotional detachment was positively related to positive affect and negatively related to health problems. Moreover, emotional detachment moderated (i.e., buffered) the negative relation between emotional demands and positive affect.
10	Atienza et al. (2020)	Vocational dancers 146 (123/22/0) 12–26 15.4 ± 2.96	Spain	Quantitative: cross-sectional survey	SVS	Amotivation positively predicted burnout and negatively predicted subjective vitality. Amotivation mediated the relationship between perfectionism and both burnout and vitality. Autonomous motivation positively predicts subjective vitality.
11	Hopper et al. (2020)	Contemporary dancer 11 (−) - -	Australian	Qualitative: focus group interviews	Making time; program specificity, dance fitness; Connecting as a company; dancer monitoring scheduling.	Psychological and physiotherapy, along with fitness training, through collaborative communities and self-manage health to maintain dancers’ well-being.
12	Rodrigues et al. (2020)	Current and former ballet dancers 40 (26/14/0) Age range 20–60 -	Portugal	Qualitative: observation and semi-structured interviews	Assessing abilities and demands; assessing needs and organizational resources; regulation strategies	Young workers aim to grow by increasing demands and enhancing personal resources. Midlife workers focus on preserving resources to maintain demands-abilities fit. Older workers compensate for resource losses by pursuing new goals or adjusting tasks.
13	Aujla et al. (2021)	Freelance dancers 282 (229/45/7) - 38.65 ± 12.68	UK	Quantitative: cross-sectional survey	18-items PWS	Grit positively correlated with the psychological wellbeing domains personal growth, purpose in life, and positive relations, but negatively with autonomy.
14	Blevins et al. (2022)	Dance students 72 (67/5/0) 16.4–27.5 18.7 ± 1.66	Australia	Quantitative: cross-sectional survey	20-items PANAS	Mindfulness is positively related to positive affect and negatively related to stress and negative affect. High mindfulness group had higher positive affect, recover and low stress compared to the low mindfulness group.
15	Collard-stokes and Irons (2022)	Dance artists and practitioners 33 (27/2/4) 25–64 44.5 ± 11.8	UK	Qualitative: Open-ended questions; focus group interviews	Barriers to wellbeing; Isolation; support networks	Independent dance artists in community health contexts face severe isolation, highlighting the importance of connection to services, professional development, and support systems for their overall well-being.
16	Lobo et al. (2022)	Collegiate dancers 177 (35/113/29) -	Philippines	Quantitative: cross-sectional survey	18-items PWS	Dance engagement positively effects students’ psychological well-being. Motivation has no moderating and mediating role in the relationship between dance engagement and psychological.
17	Wallman-Jones et al. (2022)	Ballet dancers 12 (12/0/0) 13–17 14.25 ± 1.29	Switzerland	Mixed-methods: Randomized controlled trial (8 weeks) intervention and qualitative interviews	BPNSFS	The Feldenkrais Method intervention showed no significant differences in pre-post scores for interoceptive accuracy and psychological well-being. However, interview responses reported high enjoyment, increased perceived embodied criticality in the intervention group
18	Paschali and Araújo (2023)	Dance students 10 (6/4/0) 19–23 21 ± 1.26	UK, Singapore, Greek-Cypriot, USA	Qualitative: semi-structured interviews	Definitions of health and wellbeing (H&W); Perceived enablers and barriers to H&W in high education	Dancers defined H&W as holistic, hedonic, and individual experiences. Barriers to H&W included negative body image, exhaustion, injury and peer pressure while factors like social networks and self-care supported H&W.

20-items PANAS, Positive and Negative Affect Scale (Watson et al., 1988); I-PANAS-SF, Short-Form of the Positive and Negative Affect Scale (Thompson, 2007); PANAS, Positive and Negative Affect Scale(Mackinnon et al., 1999); EAT-26, Eating Attitudes Test (Garner et al., 1987); RSES, Rosenberg Self-Esteem Scale (Rosenberg, 1965); PI, Perfectionism Inventory (Hill et al., 2004); SVS, Subjective Vitality Scale (Castillo et al., 2017); LSS, The Life Satisfaction Scale (Singh and Joseph, 1996); BPNSFS, Basic Psychological Need Satisfaction and Frustration Scale (Heissel et al., 2018); BESAA, Body-Esteem Scale for Adolescents and Adults (Mendelson et al., 2001); IPPA, Inventory of Parent and Peer Attachment (Armsden and Greenberg, 1987); AWQ, Athlete Engagement Questionnaire (Lonsdale et al., 2007); BPNSFS, Basic Psychological Need Satisfaction and Frustration Scale (Deci and Ryan, 2000); 18-items PWS, Psychological Wellbeing Scale (Ryff and Keyes, 1995).

Regarding study design, these studies included quantitative research (*n* = 12), qualitative research (*n* = 5), and mixed-methods research (*n* = 1). A variety of methodological tools were used, including cross-sectional surveys (*n* = 12), diary methods (*n* = 1), semi-structured interviews (*n* = 3), focus group interviews (*n* = 3), open-ended questions (*n* = 2) and intervention (*n* = 1). In terms of study samples, the included studies involved 2,263 participants, with 1,560 female, 643 male, and 58 participants whose gender was unspecified. Two studies reported a one-person discrepancy between total and gender counts (Atienza et al., 2020; Aujla et al., 2021). Participants ranged in age from 12 to 64 years. Most studies were conducted in the United Kingdom (*n* = 7) and the United States (*n* = 4). The remaining studies were carried out across 15 additional countries, including Canada, Japan, Belgium, Switzerland, France, and Australia.

In terms of instruments, in 13 quantitative studies, 11 different measurement tools were used. Among these measurement tools, Positive and Negative Affect Schedule (PANAS) was the most commonly used for assessing dancers’ well-being, appearing in 6 studies. Additionally, it is noteworthy that two studies used combinations of three different scales to measure dancers’ well-being. For example, Padham and Aujla (2014) measured well-being from a psychological health perspective using EAT-26, SES, and PI; Stark and Newton (2014) assessed well-being from both hedonic and eudaimonic perspectives using PANAS, BESAA, and IPPA.

Although the PANAS is widely used and valued for its strong psychometric properties, including high internal consistency, ease of administration, and sensitivity to fluctuations in affect (Crawford and Henry, 2004). This focus may not fully reflect the multidimensional nature of well-being, particularly in dance settings where factors such as identity, artistic expression, relatedness, career meaning, and self-realization are central (Pohjola, 2014; Braun and Kotera, 2021). This highlights a broader concern regarding the inconsistency of current measurement tools used in dancer well-being research.

### Appraisal of methodological quality of review studies

All included studies met the two MMAT screening questions, and were subsequently evaluated based on the five MMAT criteria for risk of bias, as presented in Table 2. Among them, six studies met all five criteria (33.3%), seven studies met the four criteria (38.8%), and five studies met three criteria (27.7%). The current systematic review identified at least one concern related to the MMAT criteria in most of the included studies. Specifically, the quality assessment found that most eligible studies (*n* = 9) have relatively small samples which can be a cause of the bias. For example, Quested and Duda (2009) had a sample of only 59 hip hop dancers, and the study by Balk et al. (2018) included 80 dance students. Additionally, six studies did not mention the examination of non-response bias or how missing data were identified and treated.

**Table 2:** MMAT quality appraisal of included studies.

Authors	Study design	Assessment criteria based on study design					Overall criteria the study met	Comments
		1	2	3	4	5		
Quested and Duda (2009)	Quantitative descriptive	✓	Cannot tell	✓	✓	✓	4 (80%)	The sample size in this study is small.
Quested and Duda (2010)	Quantitative descriptive	✓	✓	✓	✓	✓	5 (100%)	-
Padham and Aujla (2014)	Quantitative descriptive	✓	✕	✓	✕	✓	3 (60%)	Unequal sample sizes and unclear response bias.
Stark and Newton (2014)	Quantitative descriptive	✓	✕	✓	Cannot tell	✓	3 (60%)	Lack of sample diversity and unclear response bias.
Draugelis et al. (2014)	Quantitative descriptive	✓	✓	✓	Cannot tell	✓	4 (80%)	Lack of information about non-response bias.
Hancox et al. (2017)	Quantitative descriptive	✓	✓	✓	✓	✓	5 (100%)	-
Behera and Rangaiah (2017)	Quantitative descriptive	✓	✕	✓	✓	✓	4 (80%)	Selection criteria of participants are not appropriate.
Balk et al. (2018)	Quantitative descriptive	✓	✕	✕	✓	✓	3 (60%)	low sample size and some of the scales had lower internal consistencies
Atienza et al. (2020)	Quantitative descriptive	✓	Cannot tell	✓	✓	✓	4 (80%)	Small sample sizes and lack of diversity.
Aujla et al. (2021)	Quantitative descriptive	✓	✓	✓	Cannot tell	✓	4 (80%)	Lack of information about non-response bias.
Blevins et al. (2022)	Quantitative descriptive	✓	✕	✓	✕	✓	3 (60%)	low sample size and unclear response bias.
Lobo et al. (2022)	Quantitative descriptive	✓	✕	✓	Cannot tell	✓	3 (60%)	Lack of sample diversity and unclear response bias.
Cahalan and O'Sullivan (2013)	Qualitative	✓	✓	✓	✓	Cannot tell	4 (80%)	Lack of data depth, some participants may give superficial responses to open-ended questions.
Hopper et al. (2020)	Qualitative	✓	✓	✓	✓	✓	5 (100%)	-
Rodrigues et al. (2020)	Qualitative	✓	✓	✓	✓	✓	5 (100%)	-
Collard-stokes and Irons (2022)	Qualitative	✓	✓	✓	✓	✓	5 (100%)	-
Paschali and Araújo (2023)	Qualitative	✓	✓	✓	✓	✓	5 (100%)	-
Wallman-Jones et al. (2022)	Mixed method	✓	✓	✓	✓	✕	4 (80%)	Small sample size limited statistical power.

*Qualitative*: 1. Is the qualitative approach appropriate to answer the research question?; 2. Are the qualitative data collection methods adequate to address the research question?; 3. Are the findings adequately derived from the data?; 4. Is the interpretation of results sufficiently substantiated by data? 5. Is there coherence between qualitative data sources, collection, analysis and interpretation? *Mixed-methods*: 1. Is there an adequate rationale for using a mixed methods design to address the research question?; 2. Are the different components of the study effectively integrated to answer the research question? 3. Are the outputs of the integration of qualitative and quantitative components adequately interpreted? 4. Are divergences and inconsistencies between quantitative and qualitative results adequately addressed? 5. Do the different components of the study adhere to the quality criteria of each tradition of the methods involved? *Quantitative descriptive studies*: 1. Is the sampling strategy relevant to address the research question? 2. Is the sample representative of the target population? 3. Are the measurements appropriate? 4. Is the risk of nonresponse bias low? 5. Is the statistical analysis appropriate to answer the research question?

### Conceptualization of well-being in dance

In the 18 studies reviewed, only nine explicitly defined well-being, employing both quantitative (*n* = 6), qualitative (*n* = 2), and mixed methods (*n* = 1) approaches. In the literature, we identified four conceptualizations of dancers’ well-being. The first conceptualization is based on eudaimonic well-being, with five studies adopting this perspective. We found that Self-Determination Theory (SDT), particularly its Basic Psychological Needs sub-theory (BPNT), is the most dominant theoretical framework. This framework emphasizes autonomy, competence, and relatedness as foundational for human flourishing which is widely adopted in four studies (Quested and Duda, 2009, 2010; Rodrigues et al., 2020; Wallman-Jones et al., 2022) Alternatively, one study applied Ryff’s psychological well-being model, which broadens the conceptual scope to include constructs like autonomy, environmental mastery, personal growth, positive relationships, purpose in life, and self-acceptance (Ryff, 2013; Aujla et al., 2021). The second conceptualization is hedonic well-being, which was adopted by one study (Hancox et al., 2017). Their study utilized Diener’s Subjective Well-Being (SWB) framework, which focuses on pleasure attainment, happiness, life satisfaction, positive affect and low negative affect, emphasizes how people evaluate their life emotionally and cognitively (Diener, 2000). The third conceptualization treats well-being as a multidimensional construct. Stark and Newton (2014) explicitly advocate for a multidimensional definition, combining “hedonic (positive/negative affect) and eudaimonic (self-acceptance, mastery) aspects.” Paschali and Araújo (2023), within the theoretical context of Positive Psychology principles, conceptualize dancer well-being as “holistic, hedonic, and individual experiences with wide influential determinants.” Lastly, Draugelis et al. (2014) frame well-being through engagement, informed by achievement goal theory and self-concept theory, conceptualizing well-being as a context-specific psychological state characterized by vigor, dedication, and confidence.

As the included studies adopted diverse theoretical frameworks, no universal definition of dancer well-being currently exists. However, recent research tends to favor eudaimonic and multidimensional perspectives (Aujla et al., 2021; Wallman-Jones et al., 2022; Paschali and Araújo, 2023).

### Factors associated with the well-being of dancers

Factors associated with the well-being of dancers were classified in the assessed literature as (1) demographic factors, (2) motivational climate, (3) psychological factors, and (4) organizational stress and resources. The results are summarized in Table 1.

### Demographic factors

Demographic factors, including age, gender, and dance genre, showed inconsistent associations with dancers’ well-being across the included studies. Three studies explored the role of age. One study found that younger dancers reported greater fluctuations in positive affect during class Hancox et al. (2017), while two other studies reported no significant age-related differences (Padham and Aujla, 2014; Atienza et al., 2020). Gender was examined in two studies, neither of which identified significant differences in well-being outcomes between male and female dancers (Quested and Duda, 2009; Atienza et al., 2020). Regarding dance genre, one study reported that dancers in modern classes experienced smaller changes in positive affect compared to those in ballet classes (Hancox et al., 2017), whereas another found no significant differences across classical, contemporary, and Spanish dance styles (Atienza et al., 2020). These mixed findings suggest that the influence of demographic characteristics on dancer well-being remains inconclusive and may be context-dependent.

### Motivational climate

The relationship between motivational climate and dancers’ well-being is a prominent research theme. Motivational climate is commonly categorized into task-involving and ego-involving types (García-González et al., 2025). Task-involving climates emphasis on personal growth, skill mastery, and effort, consistently show strong associations with dancers’ well-being. Across studies, task-involving climates positively correlate with positive affect (r = 0.36–0.68) (Quested and Duda, 2009, 2010; Stark and Newton, 2014) and predict dancers’ engagement (vigor, dedication: *β* = 0.24–0.39) (Draugelis et al., 2014). Furthermore task-involving climates support dancers’ basic psychological needs, particularly competence and autonomy, thereby enhancing well-being (Quested and Duda, 2009, 2010). Conversely, ego-involving climates, which prioritize competition and social comparison, demonstrate weaker and inconsistent effects. Ego-involving climates correlate positively with negative affect (r = 0.33–0.45) (Quested and Duda, 2009; Stark and Newton, 2014), yet show no significant relationship with engagement (Draugelis et al., 2014). Extending these findings, Hancox et al. (2017) employing a diary-based approach, revealed that daily empowering climates (i.e., task-involving and autonomy-supportive climates) positively predict in-class positive affect via need satisfaction (*β* = 0.26). Conversely, disempowering climates (i.e., ego-involving, teacher control) were found to increase negative affect (*β* = 0.11). These findings underscore the importance of motivational climate, especially that shaped by teachers, in influencing dancers’ psychological experiences.

### Psychological factors

#### Protective and risk psychological traits

Several psychological factors with similar characteristics are closely related to dancers’ well-being, including emotional maturity, self-esteem, emotional detachment, grit, self-concept, and mindfulness. Specifically, Behera and Rangaiah (2017) found a positive correlation between dancers’ emotional maturity and life satisfaction, with self-esteem playing a key moderating role (*β* = −0.65; note: negative due to reverse scoring of emotional maturity). Research by Balk et al. (2018) indicated that emotional detachment was positively associated with positive affect (*β* = 0.23), which helped dancers maintain psychological balance and reduce concentration difficulties. Aujla et al. (2021) emphasized that grit, particularly among freelance dancers, helps maintain psychological stability in the face of career uncertainty, and is positively correlated with well-being (r = 0.21). Additionally, Blevins et al. (2022) showed that dancers with higher levels of mindfulness tend to exhibit greater positive affect, faster recovery, and lower negative affect. Other factors, such as emotional regulation strategies and resilience are crucial in enabling dancers’ well-being (Hopper et al., 2020; Rodrigues et al., 2020; Paschali and Araújo, 2023). On the other hand, self-doubt, stress, loneliness, and negative body image are barriers to dancers’ well-being (Cahalan and O'Sullivan, 2013; Paschali and Araújo, 2023).

### Dual-impact traits

Moreover, certain psychological factors such as passion, perfectionism, and motivation have a dual impact on dancer’s well-being. For example, adaptive expressions include harmonious passion, which positively predicts self-esteem (*β* = 0.37) (Padham and Aujla, 2014); self-oriented perfectionism is positively correlated with subjective vitality (r = 0.24) and autonomous motivation is positively correlated with subjective vitality (r = 0.40) (Atienza et al., 2020). In contrast, maladaptive expressions include obsessive passion, marked by internal pressure and rigidity, which positively predicts disordered eating attitudes (β = 0.27) (Padham and Aujla, 2014); socially prescribed perfectionism was negatively associated with subjective vitality (indirect effect via amotivation: IE = −0.08) and positively linked to burnout. Meanwhile, amotivation was negatively associated with subjective vitality (r = −0.43) (Atienza et al., 2020).

### Organizational stress and resources

There are critical insights into dancers’ challenges regarding organizational stress and resources. Key well-being aspects include occupational instability, intense schedules, emotional demands, physical injuries, regulatory strategies, and support systems (Cahalan and O'Sullivan, 2013; Balk et al., 2018; Hopper et al., 2020). Specifically, one study found that while many professional Irish dancers recommend their careers for benefits like travel and friendships, they also face considerable job insecurity and health risks (Cahalan and O'Sullivan, 2013). Another investigation explored contemporary dancers’ views on organizational offerings such as strength and fitness training, clinical physiotherapy, and psychology services, noting their value in maintaining health and performance despite ongoing scheduling challenges (Hopper et al., 2020). Research on professional ballet dancers further revealed that effective regulatory strategies can enhance psychological well-being by shaping how organizational resources are perceived across different life stages (Rodrigues et al., 2020). More recently, research has drawn attention to barriers to well-being and the need for stronger support networks, noting that independent dancers often feel isolated (Collard-stokes and Irons, 2022). They advocate for improved stakeholders dialogue to create clearer pathways for professional development and health support. This perspective aligns with previous findings emphasizing the importance of health literacy and emotional support networks for dance students in higher education (Paschali and Araújo, 2023). In summary, these studies collectively advocate for enhanced organizational resources and community support to alleviate stress and promote well-being in the dance profession.

## Discussion

Key findings are discussed and synthesized below concerning the extant literature. Given the methodological heterogeneity across the included studies, the findings should be interpreted with caution. The study designs varied (12 quantitative, 5 qualitative, and 1 mixed-methods), and employed diverse data collection strategies and well-being measurement tools. The sampled populations ranged from professional to non-professional dancers, with the majority of participants being female. These variations hinder meaningful comparisons across studies; therefore, our results cannot be generalized to all dance populations or across gender groups.

Firstly, regarding the conceptualization of well-being, the present synthesis revealed that dancer well-being has been conceptualized through various theoretical lenses, including hedonic (e.g., affect balance, life satisfaction), eudaimonic (e.g., personal growth, functioning), multidimensional, and engagement-based perspectives (e.g., vigor, dedication). While each framework captures important facets of well-being, the diversity also suggests a lack of conceptual consensus.

Given the unique aspects of the dance profession, such as creativity, artistry, and the demands of public performance (Docherty et al., 2025), dancer well-being may not be fully captured by a single framework. Based on the synthesis of existing literature, it is evident that dancer well-being is best understood through an integrative lens that captures both the motivational underpinnings and the psychological outcomes of dance participation. As such, we propose a tentative, dancer-centered conceptualization of well-being as a multidimensional interplay of psychological functioning, emotional states, and contextual fit. This conceptual framework that draws from Self-Determination Theory (SDT), particularly its Basic Psychological Needs sub-theory (BPNT), in combination with principles from Positive Psychology and the hedonic perspective. SDT and BPNT offer a robust structure for understanding the psychological conditions - autonomy, competence, and relatedness - that are essential for optimal functioning in the dance context (Quested and Duda, 2009, 2010; Stark and Newton, 2014). Complementary perspectives from Positive Psychology and hedonic approaches emphasize affective states (e.g., happiness, life satisfaction), as well as meaning and purpose, thereby broadening the evaluative scope of dancer well-being (Hancox et al., 2017; Paschali and Araújo, 2023). By synthesizing these theoretical perspectives, researchers can develop a multidimensional model that more accurately reflects the complex and context-specific nature of well-being in dance.

Secondly dancer’s well-being is shaped by an interplay of demographic factors, motivational climate, psychological factors, and organizational stress and resources. Among these, demographic factors showed mixed findings. Methodological differences likely explain these inconsistencies. For example, Hancox et al. (2017) used a diary-based longitudinal approach, assessing well-being (affective states) over five consecutive days before and after dance classes, finding smaller increases in positive affect in modern dance classes compared to ballet (*β* = −0.28, *p* = 0.03), and greater emotional fluctuations among younger dancers (*β* = −0.32, *p* = 0.02), heightened emotional variability typically observed during adolescence (Yang et al., 2019). In contrast, cross-sectional studies (Padham and Aujla, 2014; Atienza et al., 2020) reported no significant differences in dancers’ well-being measured as subjective vitality and self-esteem, across dance genres or age groups. These findings suggest that observed associations may vary depending on the measurement tool and study design. Future studies should employ longitudinal and cross-sectional designs to further investigate differences across age groups and dance genres, and use standardized assessment tools to enhance comparability across studies. Additionally, gender differences in well-being were not found (Quested and Duda, 2009; Atienza et al., 2020). However, this absence of gender differences should be cautiously interpreted given the overrepresentation of female dancers, which limits statistical contrasts and generalizability (Rich-Edwards et al., 2018), future research involving more balanced gender representation is necessary to clarify the relationship between gender and dancer’s well-being.

Beyond demographics, consistent evidence across hip-hop, vocational, and collegiate dancers shows task-involving climates associated with dancer’s well-being (Quested and Duda, 2009, 2010; Draugelis et al., 2014; Stark and Newton, 2014; Hancox et al., 2017). This aligns with the interplay of Achievement Goal Theory (AGT) and Self-Determination Theory (SDT) (Ames, 1992; Ryan, 2009). AGT posits task-involving climates prioritize self-referenced growth over normative comparison, creating a context where SDT’s basic needs (autonomy, competence, relatedness) are satisfied, thereby enhancing well-being (Sarrazin et al., 2001). For instance, in ballet classes, teachers can enhance dancers’ motivation by emphasizing personal progress over normative comparisons (task-involving, AGT) and by offering constructive feedback that reinforces competence (SDT) (Chua, 2016). In contrast, ego-involving climates show weaker and more inconsistent associations with well-being. This aligns tentatively with meta-analytic evidence suggesting that, among athlete samples, ego climates exert weaker effects relative to task climates (Lochbaum and Sisneros, 2024). While findings within the dance context are inconsistent, such variability may reflect nuances in sample characteristics (e.g., pre-professional vs. collegiate dancers) and methodological heterogeneity across dance well-being measures (e.g., PANAS scales vs. engagement measures) (Quested and Duda, 2010; Draugelis et al., 2014). Given these open questions, the relationship between ego-involving climates and dancers’ well-being remains an under-explored area warranting further investigation.

Furthermore, one of the salient findings was that psychological variables, including emotional maturity, self-esteem, self-concept, grit, emotional detachment, and mindfulness are protective factors critical for dancers’ well-being. Emotional maturity is a trait that enables individuals to recognize and manage their emotions (Rahmawati and Kusumiati, 2024), supporting positive emotional health, which is a crucial component of well-being. While emotional maturity and self-esteem are conceptually distinct, research indicates that high self-esteem fosters resilience and optimism, which are positively associated with well-being (Behera and Rangaiah, 2017). Self-concept, the way dancers perceive their abilities and identity, has also been shown to influence well-being significantly (Céspedes et al., 2021). This is because when self-identity is well-developed, dancers can objectively and dialectically accept themselves, enhancing their adaptability and self-regulation, promoting well-being (Draugelis et al., 2014). Grit is defined as “perseverance and passion towards long-term goals” (Duckworth et al., 2007). Gray et al. (2023) suggested that grit was an important psychological factor positively related to well-being in athletes. Our review findings were in line with the previous studies. This could be an important predictor of well-being among dancers facing high career uncertainty levels and income instability. Lastly, as a coping mechanism, emotional detachment allows dancers to maintain psychological balance in environments with high emotional demands. For instance, Balk et al. (2018) found that emotional detachment helps reduce distraction and is positively associated with positive emotions among dancers. This result echoes Balk et al. (2017), who found that high detachment is associated with improved health and well-being of athletes.

Similarly, this finding aligns with previous research demonstrating a positive link between mindfulness and well-being (Myall et al., 2023; Rehman et al., 2023). However, given the correlational nature of the current data, it remains unclear whether mindfulness directly causes improvements in dancers’ stress management, recovery, and overall well-being (Blevins et al., 2022). Therefore, future studies should consider implementing mindfulness interventions in dance populations to assess their effectiveness and identify the specific mechanisms by which mindfulness strategies influence psychological outcomes.

Also, this review found that organizational stress and resources as critical determinants of dancers’ well-being, aligning with the Job Demands-Resources Model, which posits that the balance between job demands (stressors) and resources significantly impacts occupational health and performance outcomes (Demerouti and Bakker, 2023). In dance, this balance is distinct: high demands such as job insecurity, intensive scheduling, emotional strain, and frequent physical injuries exert substantial pressure (Cahalan and O'Sullivan, 2013; Paschali and Araújo, 2023). However, organizational resources such as strength training, physiotherapy, and psychological support, as well as self-regulation strategies and social support play critical roles in alleviating these pressures, enhancing resilience, and promoting sustained well-being (Hopper et al., 2020; Rodrigues et al., 2020; Collard-stokes and Irons, 2022), mirroring the model’s emphasis on resource-driven buffering effects. These findings align with studies in athletic contexts, where personalized regulatory strategies and mental health support help sustain careers under high stress (Murdoch et al., 2024). While these insights largely stem from qualitative research, future experimental or cross-sectional quantitative studies could further validate the applicability of these constructs.

Unexpectedly, only one intervention study was identified, highlighting a significant gap in research on enhancing dancer well-being (Wallman-Jones et al., 2022). Wallman’s study, which utilized the Feldenkrais Method, a somatic mind–body approach intended to improve physical and emotional states, found no significant effects on psychological well-being dimensions. The authors attributed these null results to factors such as small sample size, brief intervention duration, and high baseline well-being, which are commonly cited as potential methodological limitations in intervention research (Boudewyn et al., 2018; Clifton and Clifton, 2019). Future studies might improve upon the design of Feldenkrais Method interventions and explore alternative approaches that have demonstrated positive effects on dancer’s mental health. For example, Mathisen et al. (2022) conducted three interactive workshops for professional dance students, addressing mental health literacy, nutrition, and performance recovery. Contextualized through case discussions and role-play, the program led to sustained improvements in mental health and nutritional knowledge, highlighting the value of arts-integrated, context-sensitive interventions. This suggests that future programs might benefit from moving beyond somatic techniques to embed psychological support within the creative and performative dimensions of dance training.

## Future direction

Given that the MMAT appraisal revealed suboptimal methodological quality in most of the included studies, particularly in areas such as nonresponse bias control and the handling of missing data and small samples. Future research should adopt methodologically rigorous designs with transparent reporting protocols to address pervasive validity threats. To overcome the inconsistencies identified in this review regarding the associations between demographic factors and well-being, researchers should also recruit larger and more diverse samples across dance genres, training levels, and cultural contexts (Uttley, 2019), thereby enhancing the reliability and generalizability of conclusion. Furthermore, enhancing gender balance by increasing the representation of male dancers is essential, as gender-balanced samples improve the generalizability and robustness of findings (Tannenbaum et al., 2019).

Second, current quantitative research relies heavily on cross-sectional designs, which limit the ability to examine temporal dynamics or infer directional relationships. To better understand how motivational climate and psychological factors influence dancer well-being over time, longitudinal and diary-based studies are needed to establish temporal precedence and explore potential causal pathways. For example, (Balk et al., 2018) acknowledged that future research should consider conducting long-term follow-ups (e.g., through monthly or daily assessments) to determine whether emotional demands precede health problems, and whether detachment consistently buffers the negative effects of high demands over time.

Third, future intervention studies should address critical gaps identified in this review. For instance, one included study found that dancers with higher mindfulness reported greater positive affect, yet no experimental design was used to test causality (Blevins et al., 2022). Randomized controlled mindfulness interventions (MI) are therefore warranted to evaluate their impact on dancer well-being. Given that MI is often integrated into broader psychological approaches such as cognitive-behavioral therapy (CBT) to enhance mental health and well-being (Sadat Tababaei Nejad et al., 2022; Gkintoni et al., 2025), which has shown effectiveness in enhancing coping skills among injured dancers (Noh et al., 2007), future studies may consider testing integrated interventions that combine MI with CBT to assess their efficacy in promoting dancer well-being. Additionally, as noted by Draugelis et al. (2014) and Hancox et al. (2017) longitudinal intervention targeting dance climate could inform teacher training strategies to support well-being. Beyond conventional approaches, future research should also innovate with arts-based psychological interventions tailored to the dance context (Mathisen et al., 2022).

Fourth, the conceptualization of well-being across the included studies remains fragmented, partly due to cultural nuances in how well-being is understood and expressed (Sollis et al., 2022). Although we have proposed a tentative, integrative framework that captures key dimensions of dancer well-being (e.g., psychological functioning, emotional states, and contextual fit), it requires further refinement. Specifically, Paschali and Araújo (2023) offer a holistic definition within higher education, additional efforts are needed to develop context-specific frameworks for diverse dance settings (e.g., professional companies, community dance groups, and cultural dance traditions). A tailored conceptualization is essential not only for theoretical coherence but also for developing valid, dancer-specific well-being measures. Such tools would facilitate meaningful cross-study comparisons and advance the field’s conceptual and empirical development.

## Limitation

This systematic review has several limitations. First, although several major databases were searched, the omission of discipline-specific sources may have restricted the coverage of relevant literature. Future reviews should consider including additional databases such as PsycINFO and Art Full Text to enhance the comprehensiveness of the evidence base. Second, although a structured search strategy was employed, some relevant studies may have been missed due to limitations in search terms. For example, some research may have conceptualized well-being using alternative terms such as “happiness” or “flourishing,” or referred to dance using different terminology. This may have resulted in unintentional exclusions or errors during the screening process. Third, while COVID-19-related studies were excluded to focus on general conditions, this choice may limit the relevance of findings in understanding dancer well-being during major pandemic. Future systematic review studies should write a separate manuscript on pandemic-related research to capture temporal variations. Finally, the lack of consistency in well-being measurement tools across the included studies weakens the strength of interpretation and comparability of findings, underscoring the need for validated, context-sensitive instruments in future research. Lastly, the included studies were characterized by increased risk of bias; therefore, the results cannot be generalized.

## Conclusion

In conclusion, this systematic review suggests that dancer well-being is increasingly conceptualized through eudaimonic and multidimensional perspectives. Building on this trend and synthesizing findings across studies, we propose a tentative, dancer-centered conceptualization of well-being as an interplay among psychological functioning, emotional states, and contextual fit. Key associated factors include demographic factors, motivational climate, psychological attributes such as self-esteem, grit, and mindfulness, as well as organizational stress and resources. However, it notes a lack of targeted intervention programs designed to enhance well-being among dancers. To strengthen research in this area, future studies should work towards a more precise conceptualization of dancers’ well-being and prioritize the development of tailored interventions that address dancers’ specific challenges. Institutions, companies, and training centers are encouraged to implement structured mental health initiatives (e.g., regular workshops on stress management), provide access to psychological services (e.g., free or subsidized counseling with therapists), and foster autonomy-supportive teaching environments (e.g., offering constructive feedback that emphasizes effort over innate ability), all of which contribute to enhancing both the physical and mental health of dancers. These conclusions should be interpreted with caution, given the methodological limitations observed in several included studies.

## Data Availability

The original contributions presented in the study are included in the article/supplementary material, further inquiries can be directed to the corresponding author.
